# Deep Learning-Based Heartbeat Detection from 3D Seismocardiography for Robust Heart Rate Monitoring

**DOI:** 10.3390/s26103254

**Published:** 2026-05-20

**Authors:** Sobuz Rana, Jukka A. Lipponen, Mika P. Tarvainen

**Affiliations:** Department of Technical Physics, University of Eastern Finland, 70211 Kuopio, Finland; jukka.lipponen@uef.fi (J.A.L.); mika.tarvainen@uef.fi (M.P.T.)

**Keywords:** accelerometers, beat detection, deep learning, heart rate monitoring, seismocardiography

## Abstract

**Highlights:**

**What are the main findings?**

- A deep learning model trained on large-scale 3D SCG signals achieves high accuracy in heartbeat detection and heart rate estimation.
- The method demonstrates robust generalization across independent datasets, including recordings from consumer-grade smartwatches, confirming its efficacy beyond controlled laboratory settings.

**What are the implications of the main findings?**

- Deep learning-based SCG analysis enables reliable, non-invasive heart rate monitoring using standard wearable accelerometers.
- Validating this approach on consumer-grade devices establishes its readiness for integration into scalable, everyday wearable health and wellness monitoring solutions.

**Abstract:**

Accurate monitoring of heart rate (HR) is critical for assessing cardiac functions in a wide range of health and wellness applications. Seismocardiography (SCG), which captures subtle chest vibrations using wearable accelerometers, provides a non-invasive and cost-effective approach for resting and nocturnal HR monitoring. This study presents a deep learning-based approach for accurate heartbeat detection and HR estimation from three-dimensional SCG signals. The model was trained on a large-scale dataset of resting SCG signals collected from 6600 subjects and evaluated on an independent cohort of 947 individuals. For short-term (≤5 min) resting SCG recordings, the model achieved robust performance in heartbeat detection (PPV: 0.979, sensitivity: 0.916, F1-score: 0.946). HR estimation showed high accuracy, with a mean absolute error (MAE) of 0.27 bpm, root mean square error (RMSE) of 1.02 bpm, and correlation of 0.996 with the reference HR. To assess real-world applicability, the model was further evaluated on 28 nocturnal recordings acquired using Apple Watch accelerometer, yielding an MAE of 1.10 bpm, an RMSE of 1.88 bpm, and a correlation of 0.982. The proposed SCG-based deep learning model demonstrates robust and highly accurate HR monitoring in both resting and nocturnal conditions, highlighting its potential for integration with consumer-grade wearable devices in a server-based analysis pipeline.

## 1. Introduction

Chronic stress is one of the most prevalent health risks in modern society, arising from occupational pressures, poor sleep quality, intense physical training, or other factors that create an imbalance between an individual’s resources and environmental demands [1,2]. Prolonged stress alters the balance of the autonomic nervous system (ANS) and, over time, increases the risk of cardiovascular diseases, metabolic disorders, and mood disturbances [1,3]. As a fundamental vital sign, resting and nocturnal heart rate (HR) provides essential insights into overall cardiovascular health and systemic physiological function [4,5]. Beyond its role as a primary parameter of cardiovascular health, HR is continuously regulated by the activities of the sympathetic and parasympathetic nervous systems, thereby acting as a key indicator of ANS balance. Typically, lower resting or nocturnal HR values indicate a well-recovered state and parasympathetic dominance, whereas an elevated HR is associated with increased sympathetic activation and physiological stress [6].

Electrocardiography (ECG) is widely known as the gold-standard method for assessing the electrical activity of the heart and person’s HR. However, the measurement of ECG necessitates the use of specialized equipment and the placement of electrodes on the skin [7]. In contrast, Photoplethysmography (PPG) is a non-invasive optical technique that measures volumetric changes in peripheral blood flow associated with cardiac cycles [7]. Ballistocardiography (BCG) represents another non-invasive approach for monitoring cardiac activity, using sensors placed under or integrated into an individual’s bed, pillow, chair, or mattress to capture subtle mechanical vibrations caused by heartbeats [8]. Seismocardiography (SCG), another noninvasive modality, quantifies cardiac-induced chest vibrations, typically using sensors embedded in chest-worn devices and smartphones. Recent technological advancements, particularly the availability of lightweight, compact, and low-noise accelerometers, have significantly enhanced the fidelity and reliability of SCG signal acquisition [9].

A wide range of techniques has been explored in the literature for heartbeat detection in BCG and SCG signals, highlighting the growing interest in personal stress or HR monitoring. Among these, template matching remains one of the most adopted approaches [10,11,12,13,14]. For SCG signals, methods such as K-means clustering and Envelope-based techniques [15], Discrete Wavelet Transform (DWT) [16], Autocorrelated Differential Algorithm [17], Hilbert Transform [18,19], Template Extraction [20], Maximum a Posteriori (MAP) [21], Hidden Markov Model (HMM) [22], and Matched Filtering [23] have been utilized. In BCG signals, heartbeat detection has been addressed using DWT- and Hilbert Transform-based methods [24], as well as Short-Time Energy analysis and Clustering approaches [25,26]. However, most existing methods implicitly assume consistent waveform morphology, overlooking the substantial inter- and intra-subject variability in BCG and SCG signals, which poses challenges for generalized and reliable heartbeat detection, and in turn, HR estimation.

Deep learning has effectively been applied to ECG, and in recent years, increasing attention has been directed toward its application in heartbeat detection and HR estimation from accelerometer-based signals. Cathelain et al. [27] introduced a heartbeat detection approach utilizing a U-Net architecture specifically designed for BCG signal processing. Similarly, a Bidirectional Long Short-Term Memory (BiLSTM) [28], and a hybrid U-Net–BiLSTM model [29] have been employed for heartbeat detection from BCG signals. For SCG signals, several deep learning architectures have also been presented, including a ResNet-based CNN [30], a U-Net-inspired SeismoNet [31], and a U-Net architecture [32] for heartbeat detection. However, only few studies have incorporated multi-channel SCG signals into their models [32]. Despite these advances, a major limitation of existing approaches lies in their reliance on relatively small datasets, which restricts the generalizability and robustness of the models across diverse subjects and real-world conditions.

In this study, we developed a deep learning model for accurate heartbeat detection and HR estimation using a large-scale dataset of three-dimensional SCG signals collected from 6600 subjects, with evaluation performed on an independent subset of 947 subjects.

The novelty of this study is threefold:

(a) The utilization of the largest SCG dataset reported to date for model development and validation;

(b) The design of a model architecture that supports three-dimensional SCG signals, demonstrating high accuracy and robustness in heartbeat detection and HR estimation;

(c) The validation of model generalizability in real-world environments using nocturnal recordings acquired with an Apple Watch accelerometer sensor.

The results of this study contribute significantly to non-invasive and unobtrusive HR monitoring using wearable accelerometer sensors, with direct relevance to various stress management, wellness and health monitoring applications.

## 2. Materials

### 2.1. Resting SCG Database

A Resting SCG database from Kubios Oy (Kuopio, Finland) was utilized in this study. According to Kubios’ privacy policy, fully pseudonymized user data may be used for research and development purposes. The database comprises daily readiness measurements collected with the Kubios HRV mobile application and the Polar H10 chest strap sensor (Polar Electro Oy, Kempele, Finland). The database includes a total of 6600 subjects, each subject having one or more recordings ranging in length from 30 to 300 s. The mean age and BMI of the subjects were 44.6 ± 14.4 years and 24.4 ± 3.7 kg/m^2^, respectively. Most of the subjects were male, comprising approximately 78.8% of the total population. The measurement data contained both beat-to-beat RR intervals (in milliseconds) and tri-axial SCG signals (x, y, and z axes) acquired with the Polar H10, positioned on the thorax inferior to the nipple line. The sampling rate of the Polar H10 accelerometer data was either 50 Hz or 200 Hz, depending on the sensor’s firmware. The RR intervals were used as a reference for the SCG-based heartbeat detection. A uniform sampling rate of 200 Hz was applied to the SCG signals using an interpolation method.

#### Training, Validation, and Test Datasets

**Figure 1:**
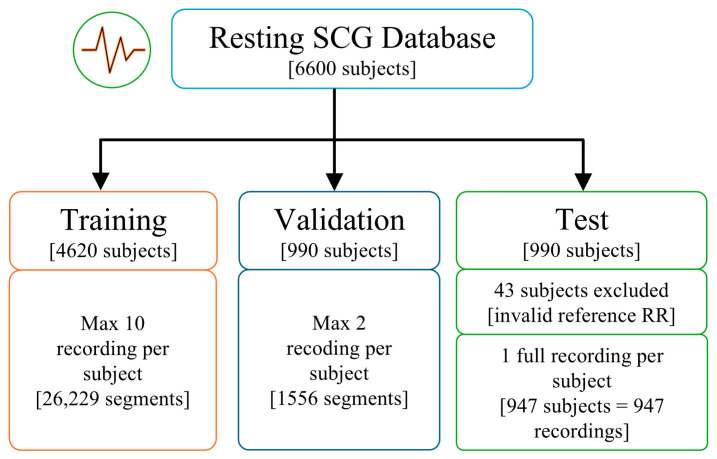
Partitioning of the resting SCG database into training, validation, and test sets with subject and segment counts.

The Resting SCG database was randomly divided into training, validation, and test sets, with 4620 subjects (70%) in the training set and 990 subjects (15%) in both the validation and test sets. For training, a segment of 4096 sample points (20.48 s) was extracted from each recording, with up to 10 recordings per subject, resulting in a total of 26,229 segments. For validation, the same segment length was utilized, but with a maximum of 2 recordings per subject, yielding a total of 1556 segments. The test set used complete recordings ranging from 30 to 300 s, with one randomly selected recording per subject. However, 43 subjects were excluded due to invalid reference RR interval values, which were confirmed through visual inspection. The final test set included 947 recordings. The composition of the training, validation, and test sets is illustrated in Figure 1.

### 2.2. Apple Watch Dataset

Additionally, we evaluated our method using the Apple Watch dataset, which is publicly available on Physionet [33]. The Apple Watch dataset comprises nocturnal physiological recordings collected from 31 participants, aged between 19 and 55 years (mean = 29.42, SD = 8.52). Signal acquisition was performed using an Apple Watch and includes tri-axial accelerometer signals sampled at 50 Hz, along with heart rate measurements derived from the watch’s built-in PPG sensor [34,35]. Three recordings were excluded from the dataset, one due to an invalid reference HR and two due to a low accelerometer signal sampling rate (~10 Hz). Signal preprocessing followed a method described in [36], which ensured that gaps between consecutive accelerometer samples and HR values did not exceed 0.05 s (implying a sampling rate > 20 Hz) and 10 s, respectively. Segments that failed to meet these criteria were excluded from the accelerometer signal. In total, 202.81 h of data from 28 subjects were processed. A total of 178.24 h (87.89%) of the data met the validity requirements, whereas 24.57 h (12.11%) were classified as invalid. Finally, all signals were interpolated to 200 Hz to align with the resting SCG database.

## 3. Methods

### 3.1. Preprocessing of Signals

**Figure 2:**
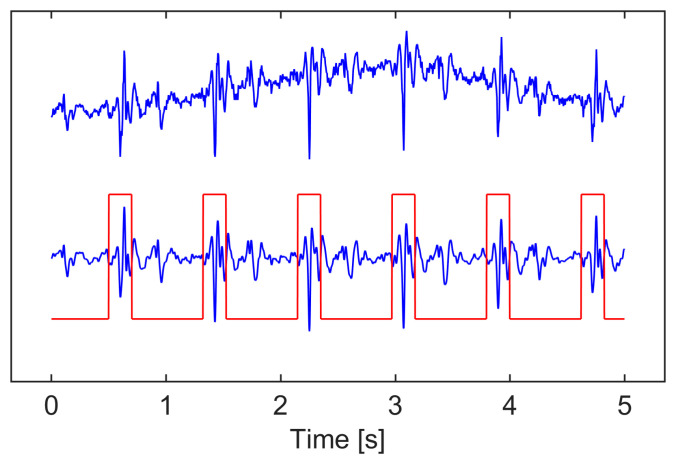
Preprocessing and heartbeat annotation of SCG signals within a 5-s window. The SCG signal is shown in blue, and the rectangular heartbeat mask is shown in red.

Raw SCG signals are susceptible to various artifacts, including low-frequency interference from respiratory motion and posture changes. To address this, a fourth-order zero-phase high pass filter with a 2 Hz cutoff frequency was designed to eliminate low-frequency noise from the signal. Furthermore, a recording-specific median-based scaling was applied to scale the signal segments, in which the median of segment-wise minimum and maximum values was used to establish stable global bounds for normalization. Figure 2 illustrates the preprocessing of the SCG signal and the annotation of heartbeats using reference RR intervals from the Polar H10 heart rate sensor within a 5-s window.

### 3.2. Model Architecture

We designed a 1D U-Net model for heartbeat detection from three-channel SCG signals, based on the original U-Net architecture developed for image segmentation [37]. The network follows an encoder–decoder architecture with symmetric skip connections to preserve spatial and temporal features throughout the model. The encoder path comprises five stages, each with two 1D convolutional layers. Each convolution is followed by a batch normalization layer and a rectified linear unit (ReLU) activation function. A max pooling layer with a stride value of 2 is used for down-sampling in each stage.

**Figure 3:**
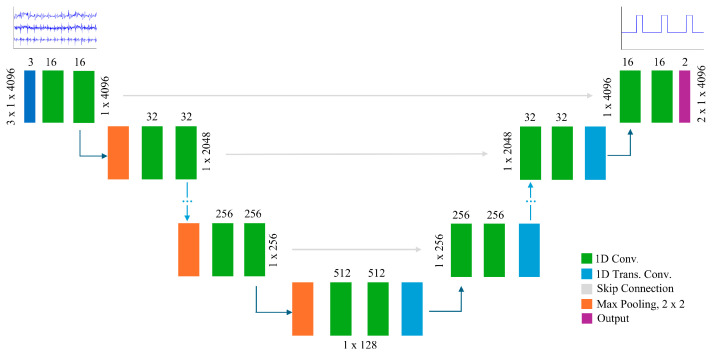
Architecture of the 1D U-Net model designed for tri-axial SCG input. The network processes 3D SCG signals and outputs a 1D mask indicating R-peak locations.

As the depth increases, the number of filters doubles at each stage. The bridge connects the encoder and decoder with two convolutional layers followed by batch normalization and a ReLU activation function. The decoder path mirrors the encoder, using 1D transposed convolutions for up-sampling, concatenating corresponding encoder outputs through skip connections, and applying similar convolutional blocks to refine the features. The final layer consists of a fully connected layer followed by a softmax function, which computes the probability distribution over the two classes, i.e., heartbeat and non-heartbeat, for each input sample point. The overall U-Net architecture is illustrated in Figure 3.

### 3.3. Model Training

The model was trained using a supervised learning approach. The input consisted of 4096 sample point segments from three-channel SCG signals (x, y, and z- axes), paired with corresponding ground truth heartbeat masks. The ground truth heartbeat masks, used for both training and validation, were derived from reference RR intervals. Specifically, each heartbeat within a segment was labeled using a binary vector based on R-peak locations extracted from the RR intervals. A symmetric window of 0.1 s was centered around each R-peak location, and all sample points within this window were labeled as heartbeats, as illustrated in Figure 2. The model’s predicted output masks were evaluated against the ground truth using a cross-entropy loss function. Training aimed to minimize this loss using the Adam optimizer, with a batch size of 128 and an initial learning rate of 10^−3^, which was reduced by a factor of 10 every two epoch if the validation loss did not improve. Training continued as long as the validation loss consistently decreased. The learning rate was progressively reduced, and training was stopped when it reached a minimum value of 10^−6^. To address class imbalance and ensure proper attention to the minority class, class weights were adjusted during training. A comprehensive summary of all key model training and implementation details is provided in Appendix A (Table A4).

#### Hyperparameter Tuning

Different configurations of key hyperparameters were explored in this study to improve the model’s performance. Specifically, a grid search was conducted over the network depth, convolutional filter size, and the number of filters in the first encoder layer, using initial values drawn from the existing literature [27,29,38]. The search space was further expanded to identify an optimal set of hyperparameters, and the best configuration was selected based on validation loss. The final model utilized a depth of 5, 16 filters in the initial encoder layer, and a convolutional filter size of 30.

### 3.4. Heartbeat Detection

To precisely identify heartbeat locations from the predicted masks, the masks were interpolated to a sampling rate of 1000 Hz, and a threshold of 0.8 was applied to retain only those masks with high prediction confidence. This threshold was selected empirically by evaluating a range of threshold values and choosing the value that provided the best balance between retaining true heartbeat regions and suppressing low-confidence false detections. Following this, two filtering criteria were introduced to refine the predicted mask. First, each predicted mask segment was required to have a minimum width of 160 sample points, equivalent to 80% of the width of the corresponding reference mask, to exclude segments unlikely to represent actual heartbeats. Second, to ensure physiological plausibility and avoid detecting duplicate or ectopic beats, a minimum separation of 0.55 s was enforced between consecutive predicted heartbeat masks. Finally, the mean index of each remaining mask was computed to determine the precise temporal location of the predicted heartbeat.

### 3.5. Heart Rate Estimation

Heart rate was estimated by calculating the time intervals between successive heartbeats. The inter-beat interval (IBI) data were then pre-processed to ensure reliable HR estimation. Firstly, a signal quality detection algorithm of the Kubios HRV Scientific software (Version: 4.2.0) was applied to the IBI data to identify corrupted data segments to be excluded from HR estimation. Secondly, the IBI data were processed using a beat correction algorithm, which detects and corrects missed, misaligned, or extra beats by applying interpolation-based adjustments to ensure physiologically plausible intervals, as described in [39]. HR was then computed in a sliding window with a fixed window size of 20 s. HR was estimated if the effective length of good-quality IBI data exceeded 50% of the window length. The effective length was calculated by summing the RR intervals that were neither excluded by the quality algorithm nor adjusted by the beat correction method and then dividing this sum by the length of the analysis window. This approach ensured that only data of sufficient quality and quantity were used for reliable HR estimation. HR was then estimated using the formula:

HR [bpm] = 60/mean (IBI) [seconds]

### 3.6. Statistical Analysis

The accuracy of heartbeat detection was evaluated by comparing the temporal location of predicted heartbeats against reference heartbeats using a specified tolerance of 0.1 s. A predicted heartbeat was classified as a true positive (TP) if it occurred within the tolerance window of a reference heartbeat. Any reference heartbeat not matched to a predicted heartbeat within this window was considered a false negative (FN), indicating a missed detection. Conversely, any predicted heartbeat that did not correspond to a reference heartbeat within the tolerance interval was classified as a false positive (FP), representing an incorrect detection. This one-to-one matching between predicted and reference heartbeats provides a clear assessment of detection performance in terms of correctly identified, missed, and wrongly detected heartbeats. Model performance was then quantified using positive predictive value (PPV), sensitivity, and F1-score, based on the total number of TP, FP, and FN from the test dataset. Heart rate estimation accuracy was assessed by comparing the estimated HR to the reference HR from the same segment using mean absolute error (MAE), root mean square error (RMSE), and the Pearson correlation coefficient. The agreement between the estimated and reference HR was further evaluated using Bland–Altman methodology.

### 3.7. Movement Artifacts Detection

Although the dataset is labeled as the resting SCG database, it cannot be definitively confirmed that all recordings were acquired while subjects were in a true supine resting position, as instructed by the Kubios HRV mobile application. To evaluate the performance of our model under movement-free conditions, a spectral energy-based approach utilizing the Short-Time Fourier Transform (STFT) was employed to identify and eliminate motion artifacts from the signals. This method, adapted from a previously established approach [36], divides SCG signals into 10-s segments. A segment is classified as an artifact if its spectral energy exceeds five times the minimum spectral energy observed within the same recording. It should be emphasized that this procedure was implemented solely to evaluate the performance of the proposed model under both optimal (movement-free) and non-optimal conditions.

## 4. Results

### 4.1. Performance Evaluation on Resting SCG Database

#### 4.1.1. Heartbeat Detection Analysis

The performance of the model was evaluated on the test set containing 947 recordings, totalling 50.70 h and 190,205 annotated heartbeats. The model achieved a PPV of 0.979, a sensitivity of 0.916, and an F1-score of 0.946 as presented in Table 1, demonstrating its robust effectiveness in heartbeat detection.

**Figure 4:**
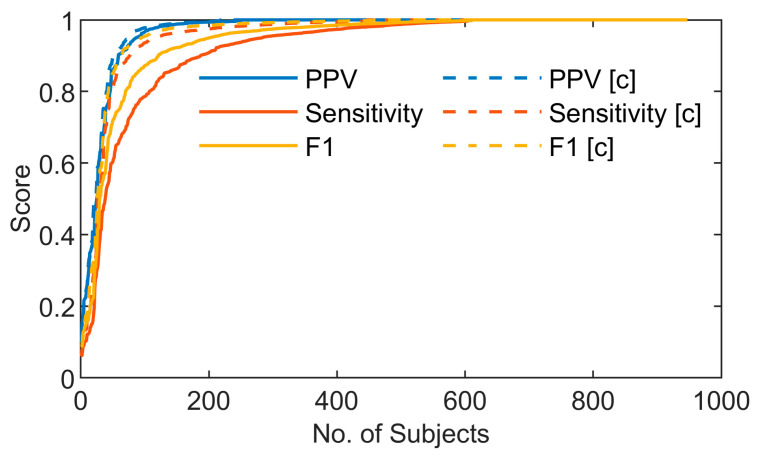
Heartbeat detection performance metrics (PPV, Sensitivity, and F1-score) across all subjects before (solid line) and after (dashed line) movement artifact correction.

To further evaluate the performance of the model on clean and artifact-free SCG signals, predicted and reference heartbeats in corrupted segments were removed using the method described in Section 3.7. This process enabled us to assess to what extent missed beats were associated with motion-corrupted segments, which provides an insight into the model’s performance under optimal signal conditions. The movement artifact detection method identified 10.32% of the total recording time, corresponding to 5.23 h, as corrupted by motion artifacts. After removing these corrupted segments, the model achieved a PPV of 0.984, a sensitivity of 0.966, and an F1-score of 0.975 as summarized in Table 1. Figure 4 illustrates a subject-wise comparison of heartbeat detection performance, measured using PPV, sensitivity, and F1 score before and after removing segments corrupted by movement.

#### 4.1.2. Estimation of Heart Rate

**Figure 5:**
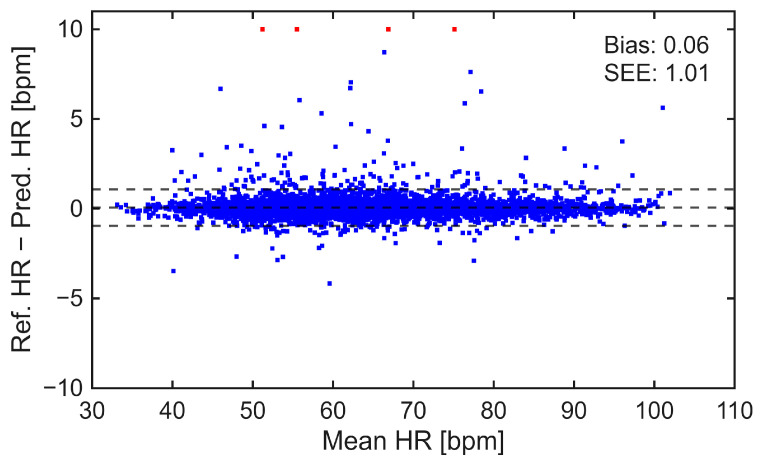
Bland–Altman analysis for HR estimation illustrating the agreement between estimated and reference HR with mean bias and standard error of estimation (SEE); outliers (in red) are clipped at ±10 bpm for visualization. The middle dashed line represents the mean bias, while the upper and lower dashed lines represent the limits of agreement (mean ± SD).

Heart rate estimation was performed based on the model’s heartbeat detection. A total of 9013 segments, each with a duration of 20 s, were analyzed to estimate HR. Of these, 16.99% (*n* = 1531) were not analyzed due to insufficient effective signal length within the 20-s window. The model achieved an MAE of 0.27 bpm, an RMSE of 1.02 bpm, and a correlation of 0.996 with the reference HR, as presented in Table 2. The agreement between estimated and reference HR was further examined using Bland–Altman analysis, shown in Figure 5. The results indicated a mean difference of 0.06 bpm, with limits of agreement (LoA) ranging from −0.95 to 1.07 bpm.

### 4.2. Performance Evaluation on Nocturnal HR Estimation

To assess performance on real-world signals acquired from a consumer-grade wrist-worn device, the model was further evaluated on the Apple Watch dataset.

**Figure 6:**
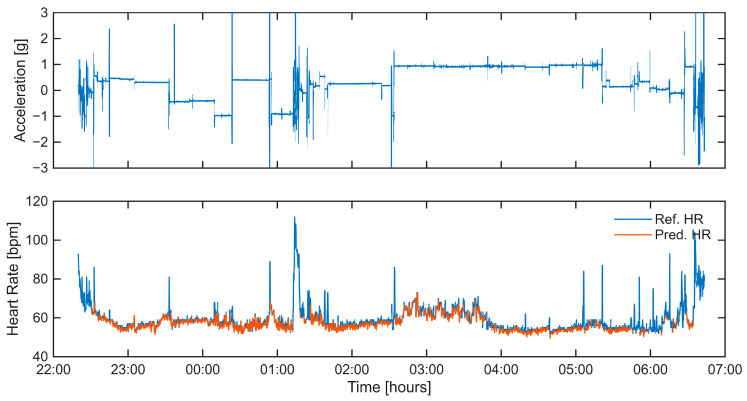
*Z*-axis acceleration signal from a representative nocturnal recording together with the corresponding reference HR and the estimated HR, demonstrating their agreement over time.

Across the entire dataset, a total of 144.22 h (80.91%) were analyzable, and 34.02 h (19.09%) were non-analyzable due to insufficient effective signal length. The model demonstrated strong performance, achieving an MAE of 1.10 bpm, an RMSE of 1.88 bpm, and a correlation of 0.982. Figure 6 illustrates the results from a representative subject, for whom the model achieved an MAE of 0.95 bpm, an RMSE of 1.14 bpm, and a correlation of 0.971. The total duration of the recording shown in Figure 6 included in the analysis was 7.96 h, of which 6.20 h were analyzable, and 1.77 h were non-analyzable because of low effective signal length.

As shown in Figure 6, at approximately 01:14 a.m., the subject either moved or changed posture, leading to a rise in HR. During this period, the model failed to estimate HR accurately due to motion-induced artifacts corrupting the accelerometer signal. Similar short disruptions can be observed at other timepoints where the subject moved or altered posture.

## 5. Discussion

This study introduces a high-performing deep learning model for heartbeat detection and HR estimation using three-dimensional SCG signals. The model was evaluated on short resting SCG measurements from 947 individuals acquired with a Polar H10 chest-strap sensor, as well as on 28 nocturnal recordings collected with an Apple Watch accelerometer. The model demonstrated good HR estimation performance in resting conditions (MAE: 0.27 bpm, correlation: 0.996) and in nocturnal recordings (MAE: 1.10 bpm, correlation: 0.982). These results indicate that the proposed deep learning-based approach maintains high accuracy across both short-term resting and nocturnal monitoring conditions, supporting its suitability for unobtrusive HR monitoring using wearable accelerometers with server-based analysis.

Previous studies have reported high heartbeat detection performance using both traditional and deep learning approaches, when evaluated under controlled resting conditions, a detailed comparison of these methods is provided in Appendix A (Table A1 and Table A2), summarizing dataset characteristics, input modalities, and performance metrics. Reported PPV and sensitivity values for traditional methods range from 0.93 to 0.99 and 0.89 to 0.99, respectively [11,12,15,18,19,20]. Among deep learning approaches, Craighero et al. [32] reported a PPV of 0.95. Similarly, Duraj et al. [30] reported a PPV of 0.969, while Suresh et al. [31] achieved a higher PPV and sensitivity of 0.98 but evaluated their model on the same subjects used for training. In the present study, the model achieved an overall PPV of 0.979 and sensitivity of 0.916 on the resting SCG test dataset, and 0.984 and 0.966, respectively, for non-movement segments, which is comparable to controlled resting conditions. These results indicate that, under controlled conditions, both traditional and deep learning methods can achieve very high and comparable heartbeat detection performance. However, previous studies have typically relied on small cohorts or non-independent evaluation protocols. In contrast, the proposed method was evaluated on a large-scale, independent test set, supporting improved generalizability of the findings.

For HR estimation, the proposed model achieved high accuracy and temporal stability. This is reflected in the low MAE of 0.27 bpm and RMSE of 1.02 bpm, as well as a near-perfect correlation of 0.996 with the reference HR. Furthermore, the Bland–Altman analysis indicated a minimal bias of 0.06 bpm and SEE of 1.01 bpm, which suggests a good agreement between the estimated and reference measurements. Outlier analysis provides additional insight into the robustness of the model. A total of 4 outliers were identified, where the difference between reference and estimated HR exceeded 10 bpm. Visual inspection revealed that these cases were associated with poor signal quality: one subject’s SCG recording lacked visible heartbeats, while the remaining three were severely corrupted by motion artifacts.

In comparison to prior work, the proposed approach demonstrates clear improvements, a detailed comparison of HR estimation performance across methods is provided in Appendix A (Table A3). Earlier studies, such as [22], employed traditional methods including HMM, envelope, and spectral-based analysis, which yielded lower accuracy relative to this study. Only few prior studies have explored deep learning-based HR estimation from resting SCG signals. Among them, Lee and Whang [40] demonstrated relatively high MAE (1.67 bpm), RMSE (2.60 bpm), and strong correlation (0.982), while Chen et al. [41] achieved a similar high correlation (0.98) but did not report explicit error metrics. In contrast, this study shows a substantial improvement in accuracy, as indicated by a lower MAE, while maintaining a similarly high correlation with the reference HR. Although all the mentioned approaches achieved strong correlation, it is essential to note that both previous studies evaluated their models on a relatively small test dataset.

**Figure A1:**
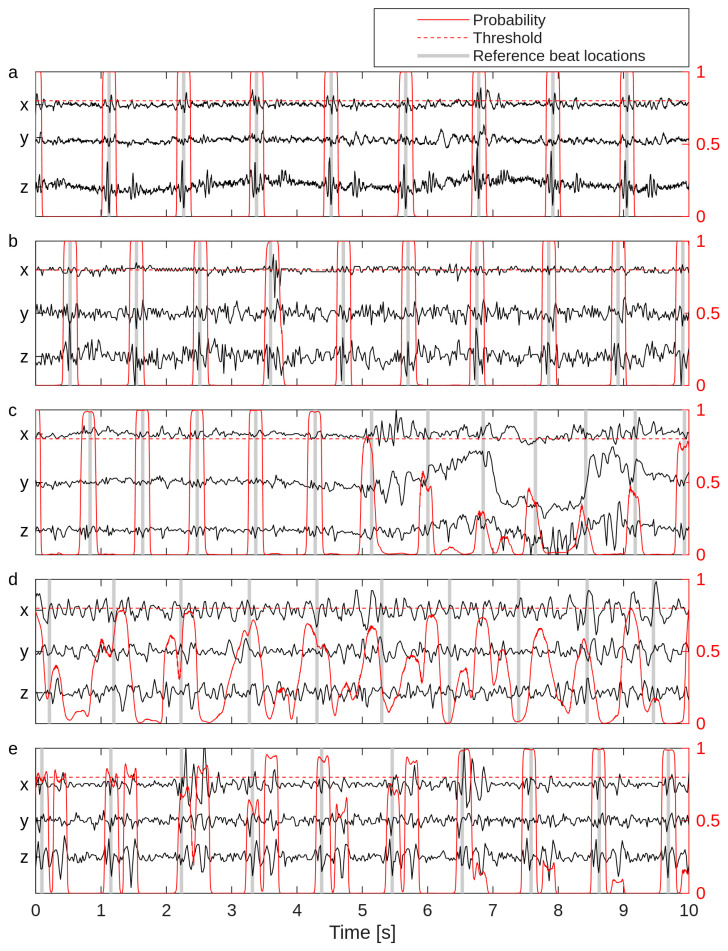
Examples of model outputs under diverse SCG scenarios: (**a**) high-quality signal, (**b**) noisy signal with successful detection, (**c**) motion-affected segment, (**d**) signals where beats are not visible, and (**e**) signal where heartbeats exhibit a double-peaked waveform, causing duplicate detections.

Additionally, to investigate cases with poor model performance, SCG recordings from 106 subjects with model sensitivity below 80% were analyzed. The findings indicate that the predominant factor contributing to reduced sensitivity was motion artifacts, observed in 87 recordings (92.22%). Notably, 60 of these cases were successfully identified by the artifact removal method described in Section 3.7. In addition to motion-related disturbances, signal quality issues were also evident. In 14 subjects (13.21%), heartbeats were not clearly visible within the SCG recordings. Furthermore, 5 subjects (4.72%) exhibited atypical signal morphology, characterized by dual acceleration patterns within a single heartbeat cycle in one or more channels. In such cases, the model demonstrated inconsistent behavior, variably detecting the first peak, the second peak, or an intermediate point as the representative heartbeat. Examples of high-quality signals and common detection errors are visualized in Appendix A (Figure A1).

To assess the generalizability of the proposed model, its performance was evaluated on an independent nocturnal dataset acquired using the Apple Watch. This evaluation aimed to determine whether heartbeat representations learned from large-scale, multiaxial SCG recordings could generalize to distal cardiogenic vibration signals acquired at the wrist using consumer-grade accelerometers. Although the Apple Watch accelerometer recordings differ from chest-based SCG in their biomechanical origin and signal morphology, they contain cardiogenic vibration patterns commonly associated with distal BCG. While SCG and BCG originate from different biomechanical mechanisms, both reflect the same underlying cardiac cycle and, therefore, preserve heartbeat-related temporal information [42]. Previous studies have demonstrated that wrist mounted accelerometers can capture meaningful cardiogenic signals for heartbeat and cardiac timing estimation, particularly during low-motion conditions such as sleep [36,43].

Despite the differences in signal acquisition modalities, the model maintained robust performance, achieving an MAE of 1.10 bpm, an RMSE of 1.88 bpm, and a correlation of 0.982 with the reference HR. These results indicate that the model is capable of effectively capturing HR dynamics in extended, real-world monitoring scenarios such as overnight recordings. A closer examination of an individual recording, shown in Figure 6, reveals that the model consistently tracked HR trends throughout the night, except for segments affected by movement and postural changes, which introduced motion artifacts and corrupted the signal. This observation underscores a fundamental limitation of accelerometer-based HR estimation: reliable performance is contingent on signal integrity, and accurate estimation remains challenging during periods of significant motion where the accelerometer signal becomes corrupted.

A comparative evaluation was conducted against a traditional approach proposed by Moebus et al. [36], which was evaluated on the same Apple Watch dataset. Their method achieved an MAE of 1.68 bpm, an RMSE of 3.38 bpm, and a correlation of 0.64. In contrast, the proposed model achieved substantially improved performance across all metrics. It is also noteworthy that their method employed a 5-min window for HR estimation, whereas the present study utilized significantly shorter 20-s windows while still attaining higher accuracy. These results highlight the advances of deep learning-based methods over traditional approaches for accelerometer-based HR estimation.

The observed generalization to nocturnal Apple Watch recordings can be attributed to the characteristics of both the training data and the proposed network architecture. Although the model was trained using short resting 3D SCG recordings, the use of multiaxial accelerometer signals, together with a large-scale training dataset, exposed the network to substantial inter-subject and inter-axis variability arising from differences in sensor orientation, body anatomy, respiratory activity, and natural physiological fluctuations. Consequently, the model learned robust temporal representations of recurring cardiomechanical activity associated with the cardiac cycle rather than relying solely on fixed SCG waveform morphology. This is particularly relevant because the accelerometer signal acquired from the wrist during sleep is physiologically related to wearable BCG, which, despite having different morphology from SCG, still preserve heartbeat-related temporal dynamics. The low errors observed in the Apple Watch recordings suggest that, facilitated by its multiscale feature extraction capability, the network has learned robust heartbeat-related temporal patterns that are preserved across different cardiomechanical sensing modalities. Nevertheless, important limitations remain. Wrist-worn accelerometer signals exhibit lower signal-to-noise ratios and substantially greater motion sensitivity than chest-mounted accelerometer recordings. Signal quality may vary considerably depending on hand movement, sleeping posture, watch fit, and body anatomy. As demonstrated in Figure 6, postural transitions and movement artifacts can corrupt the cardiogenic signal and degrade HR estimation accuracy. This limitation is consistent with previous studies investigating motion artifacts in wearable sensing systems [44].

The performance of the proposed U-Net stems from its encoder–decoder architecture with skip connections, which enables effective multi-scale feature extraction for accurate heartbeat localization. This aligns with prior work demonstrating the suitability of U-Net–based architectures for beat detection in SCG and BCG signals [27,31,32]. We also evaluated alternative architectures, including a U-Net-BiLSTM variant with a Bi-LSTM layer in the bottleneck and a plain ResNet-style network; however, these approaches consistently yielded lower performance compared to the proposed U-Net model. To evaluate the benefit of three-channel SCG information, we compared single-channel and three-channel versions of the proposed U-Net model. Although the overall increase in mean F1-score was modest (from 0.944 to 0.946), a more detailed per-recording evaluation revealed meaningful improvements in more challenging cases. The three-channel model outperformed the single-axis configuration in 33 recordings, with substantial gains exceeding 0.2, including improvements greater than 0.3 in 18 recordings. These findings indicate that 3D SCG input enhances model robustness, particularly in conditions characterized by lower signal quality or greater morphological variability.

A significant strength of this study is the scale and diversity of the dataset, which included a notably large number of subjects for both the training and testing phases. Importantly, the training and testing datasets consisted of different individuals, ensuring a rigorous evaluation of model generalizability. Another strength is that the SCG signals were collected in uncontrolled environments, which naturally included motion artifacts. The model’s robust performance under these conditions demonstrates resilience to real-world variability. In addition, the resting SCG database used a validated reference HR sensor, the Polar H10, further supporting its reliability. However, in the Apple Watch dataset, the reference HR was obtained from an optical sensor rather than ECG. Optical sensors estimate HR indirectly by detecting changes in blood volume using light, making them more susceptible to errors caused by motion artifacts and ambient light interference. Consequently, the reported evaluation metrics may either underestimate or overestimate the model’s true performance. The developed model is capable of detecting heartbeats from SCG signals only in segments with minimal movement, such as breathing. Future models incorporating motion compensation or multi-modal sensor fusion may enable reliable performance under more dynamic conditions. In addition, the proposed model is not optimized for edge deployment, as the intended use case assumes that SCG signals are recorded with wearable or mobile devices and processed in a server-based environment.

## 6. Conclusions

In this study, a deep learning model was developed and evaluated for heartbeat detection and HR estimation using resting SCG signals. The model achieved high accuracy across both short-term resting measurements and full-night sleep recordings collected with consumer-grade wearables, maintaining strong performance despite variations in accelerometer sensor placement and recording conditions. These results underscore the potential of SCG-based monitoring as a reliable and scalable solution for resting HR monitoring in wearable sensor technologies.

## Figures and Tables

**Table 1:** Quantitative evaluation of heartbeat detection performance on resting SCG, both before and after excluding motion artifacts, expressed in terms of positive predictive value (PPV), sensitivity, and F1-score.

Motion Artifacts	PPV	Sensitivity	F1-Score
Not excluded	0.979	0.916	0.946
Excluded	0.984	0.966	0.975

**Table 2:** Heart rate estimation performance summarized using mean absolute error (MAE), root mean square error (RMSE), and correlation coefficient.

Dataset	MAE	RMSE	Correlation
Resting SCG	0.27	1.02	0.996

## Data Availability

The raw data for the resting SCG database cannot be shared, as participants did not consent to public distribution under privacy policy. The Apple Watch dataset is publicly available at PhysioNet.

## Appendix A

**Table A1:** Summarizes studies that employ traditional signal processing methods for heartbeat detection using BCG and SCG signals. Performance is reported in terms of positive predictive value (PPV), sensitivity, and F1-score where available. Variations in reported ranges reflect differences in datasets and evaluation protocols.

Study	Methods	Signal	Number of Subject	Separate Test Set	PPV	Sensitivity	F1-Score
Cathelain et al. (2019) [10]	Template matching	BCG	10	No	96.8%	95.6%	-
Parlato et al. (2025) [11]	Template matching	SCG	49 (H), 77 (P)	No	97.7% 94.9%	97.8% 85.2%	-
Cinotti et al. (2025) [12]	Template matching	SCG	10	No	93.3%	89.3%	-
Centracchio et al. (2023) [13]	Template matching	SCG	77 (VHD)	No	79.0–96.0%	82.0–97.0%	-
Cocconcelli et al. (2020) [14]	Template matching	SCG	20, 13	Yes	98.6% 97.9%	98.5% 99.1%	-
Kaisti et al. (2019) [15]	Clustering and Envelope	SCG	29, 12 (CAD)	Yes	99.6% 76.5–92.8%	99.9% 75.6–91.7%	-
D’Mello et al. (2019) [17]	Autocorrelated differential algorithm (ADA)	SCG GCG	25	No	99.68%	96.57%	-
Chudhary et al. (2020) [18]	Hilbert transform and Envelope	SCG	20, 10	Yes	97.4% 96.4%	97.3% 96.7%	-
Jafari Tadi et al. (2016) [19]	Hilbert transform	SCG	29	No	96.0%	95.8%	-
Mora et al. (2020) [20]	Variational autoencoder (VAE)	SCG	20	No	98.6%	98.5%	-
Schipper et al. (2024) [21]	Maximun a posteriory (MAP)	SCG	147	No	98.0–100%	79.3–88.9%	-
Proposed	1D U-Net	SCG SCG*	6600	Yes	97.9% 98.4%	91.6% 96.6%	94.6% 97.5%

H: Healthy; P: Pathological; VHD: Valvular heart disease; CAD: Coronary artery disease (CAD); SCG*: Movement free SCG signal.

**Table A2:** Presents studies that utilize deep learning-based models for heartbeat detection. The reported metrics include positive predictive value (PPV), sensitivity, and F1-score.

Study	Model	Signal	Number of Subject	Separate Test Set	PPV	Sensitivity	F1-Score
Cathelain et al. (2020) [27]	U-Net	BCG	40	Yes	92.1%	80.9%	85.7%
Mai et al. (2022) [29]	U-Net-Bi-LSTM	BCG	43	Yes	96.89–98.00%	-	-
Duraj et al. (2022) [30]	CNN	SCG	20	Yes	96.9%	99.9%	98.1–99.2%
Suresh et al. (2020) [31]	SeismoNet	SCG	20	No	98.0%	98.0%	-
Craighero et al. (2024) [32]	U-Net	SCG	72	Yes	83.0–95.0%	82.0–98.0%	81.0–96.0%
Proposed	1D U-Net	SCG SCG*	6000	Yes	97.9% 98.4%	91.6% 96.6%	94.6% 97.5%

SCG*: Movement free SCG signal.

**Table A3:** Compares the performance of different methods for HR estimation. Metrics include mean absolute error (MAE), root mean square error (RMSE), and correlation coefficient.

Study	Model	Signal	Number of Subjects	Separate Test Set	MAE	RMSE	Correlation
Wahlström et al. (2017) [22]	HMM, Envelope, Spectral	SCG	66	Yes, Only 1	0.56–0.78 5.00–6.86 7.06–9.69	-	-
Moebus et al. (2025) [36]	Traditional	BCG ACC	42, 32 (AW)	Yes	0.88 1.68	2.24 3.38	0.81 0.64
Lee and Whang (2018) [40]	VGG-16, VGG-19, Ensemble	SCG	30	Yes	1.67 4.06 2.17	3.09 6.47 3.80	0.982 0.946 0.988
Chen et al. (2021) [41]	Bi-LSTM	SCG	20	No	1.3	-	0.98
Proposed	U-Net	SCG ACC	6600, 32 (AW)	Yes	0.27 1.10	1.02 1.88	0.996 0.982

HMM: Hidden Markov model; AW: Apple Watch dataset; ACC: Acceletometer signal.

**Table A4:** Summary of model training settings and optimal hyperparameters.

Parameter	Value
Input length	4096 samples
Number of channels	3 (x, y, z)
Optimizer	Adam
Initial learning rate	1 × 10^−3^
Learning rate schedule	Reduce × 0.1
Minimum learning rate	1 × 10^−6^
Batch size	128
Loss function	Weighted cross-entropy
Class weights	[0.6237, 2.6140]
Max epochs	20
Early stopping	Validation loss increases (patience = 2)
Network depth	5
Initial filters	16
Kernel size	30
Framework	MATLAB (R2024b) Deep Learning Toolbox
Hardware	NVIDIA GeForce RTX 3080 10 GB Memory
Training time	~30 min
